# Evaluation of intra-ovarian platelet-rich plasma administration on oocytes-dependent variables in patients with poor ovarian response: A retrospective study according to the POSEIDON criteria

**DOI:** 10.1186/s12958-021-00826-w

**Published:** 2021-09-08

**Authors:** Marzieh Farimani, Arash Nazari, Shahrzad Mohammadi, Roghayeh Anvari Aliabad

**Affiliations:** 1 Endometrium and Endometriosis Research Center, Hamadan University of Medical Sciences, Hamadan, Iran; 2 School of Medicine, Hamadan University of Medical Sciences, Hamadan, Iran; 3 School of Medicine, Iran University of Medical Sciences, Tehran, Iran; 4 Department of Gynecology, School of Medicine, Hamadan University of Medical Sciences, Hamadan, Iran; 5 Endometrium and Endometriosis Research Center, Fatemieh Hospital, Pasdaran Street, P.O. Box, 89971-65177 Hamadan, Iran

**Keywords:** Female infertility, Poor ovarian response, Platelet-rich plasma, POSEIDON criteria

## Abstract

**Background:**

Poor ovarian response (POR) is among the common findings in infertile women with no significant underlying condition. The aim of this study was to investigate the intra-ovarian potential of platelet-rich plasma (PRP) administration on oocytes-dependent variables in the POR women grouped according to the POSEIDON criteria.

**Methods:**

This retrospective study was performed on POR women with no underlying condition who have undergone intra-ovarian PRP injection. As well as patients’ age, the number of total and MI, MII, and GV oocytes were extracted from the files. The laboratory variables including anti-mullerian hormone (AMH), follicle-stimulating hormone (FSH), luteinizing hormone (LH), and estradiol were also gathered. In order to reduce any bias due to the possible differences in kits or devices, a single laboratory with the highest number of cases was selected and others were excluded from the study. Then, the included cases were grouped into four according to the POSEIDON criteria and analyzed for the mentioned variables by SPSS, version 25. The statistical significance level was set as *P*-value < 0.05.

**Results:**

From 383 cases, a total number of 96 women were enrolled in this study. According to the POSEIDON criteria, group 4 (Age ≥ 35 years, AMH < 1.2 ng/mL) with the ratio of 56/96 (58.3%) had the highest prevalence among others. As the analyses showed, changes in the laboratory variables (LH, FSH, AMH, and estradiol) were not significant in almost all the groups following the intervention. Regarding the total oocytes number, PRP administration caused a significant increase in the total number in all the groups (all *P* < 0.05). Also, the number of MII oocytes was significantly increased following the treatment in all groups except for group 2 (Age ≥ 35 years, AMH ≥ 1.2 ng/mL; all *P* < 0.05). Of 96 cases, 14 (14.6%) got clinically pregnant following assisted reproductive techniques which this number were significantly differed among the groups (*P* = 0.002).

**Conclusion:**

This study showed that PRP treatment was effective on total and MII oocyte numbers in the patients with POR, however, further studies are required.

## Background

Aging causes different changes in the physiology of the human body and is associated with increased the risk of infertility in females. Over the course of time, ovaries experience a decrease in their follicular quantity (ovarian reserve) also knowns as poor ovarian reserve [1]. Nowadays, assisted reproductive techniques (ART) are being widely used in the clinic to overcome this problem. However, some patients do not respond to the stimulators administrated (such as gonadotropin) before ART which has been described as poor ovarian response (POR) [2–4]. Despite age, so far, different laboratory tests and ultrasound investigations have been used as routine techniques to predict the probability of ART success in these cases. Also, anti- Mullerian hormone (AMH), follicle-stimulating hormone (FSH), luteinizing hormone (LH), inhibin, and estradiol levels are among the helpful laboratory tests for this aim. However, invasive methods such as ovarian biopsy have also been mentioned by studies [1]. So far, a notable number of investigations have tried to define POR according to the clinical and para-clinical status of cases as a standard definition [3]. Among these definitions, certain ones such as the Bologna criteria [5] has been used in many trials and studies. Also, for evaluation of prognosis for women undergoing ART and due to the importance of different approaches in these patients, a new definition (as well as a category/grouping) has been presented called POSEIDON criteria [3, 6].

So far, platelet-rich plasma (PRP) has been used in many trials for accelerating the healing of acute [7] and chronic [8] wounds, plastic surgeries [9], tendinopathies [10] and other regenerative goals. A few years ago, we used intrauterine PRP injection in patients with recurrent implantation failure (RIF) which led to noticeable findings [11]. Then, we aimed to use this autologous product for women with poor response to gonadotropin stimulation as well [12]. Nowadays, PRP is used more widely in reproductive medicine due to its regenerative potentials; however, not enough data is available on this issue [13]. One of the new strategies for facing primary ovarian insufficiency is PRP therapy. A report on 23 women with primary ovarian insufficiency has shown that PRP could be a proper treatment option for these patients [14]. Recently, PRP therapy has been used as a possible treatment for 17 women diagnosed with poor ovarian response (POR) which seemed potent enough to be a possible future treatment according to the obtained results [15]. After the first live birth following PRP usage in a woman with primary infertility [16], we aimed to investigate the potential of intra-ovarian PRP injection in women with POR and comparing the obtained results in the groups categorized according to the POSEIDON criteria.

## Methods and patients

### Study design and ethics

This retrospective study (on original data) has been performed in Omid Clinic (Hamedan, Iran) from April 2018 to April 2020. The current study was approved by the Medical Ethics Committee of Hamedan University of Medical Sciences (Hamedan, Iran) with the IRB number of IR.UMSHA.REC.1399.725. The inclusion criteria for this study was considered as any POR (according to the Bologna [17] criteria) woman undergone intra-ovarian PRP injection. Lack of follow-ups and incomplete laboratory results were considered as the primary exclusion criteria. In order to reduce any bias due to possible differences in kits/devices/operators, a single laboratory with the highest number of cases was selected and cases with results from elsewhere were excluded from the study. Diseases/disorders affecting the chance of fertility were also among the exclusion criteria but since they were already checked at the time of admission (by the clinic), no further checking was done by the current study.

### Platelet-rich plasma and intervention

The clinic followed the exclusion criteria for PRP preparation which were platelet count < 10^6^/ml, hemoglobin < 10 mg/dL, and any contra-indication for peripheral venous access [18]. PRP was prepared according to the already reported similar protocols [11, 12, 16]. The Shanghai protocol [19] was used for ovarian stimulation. Right after the first follicular puncture, the intra-ovarian PRP injection (2 ml) was performed under ultrasound guide followed by the second puncture for the second stimulation. AMH, LH, FSH, and estradiol levels were measured before the intervention (baseline) as well as the after two menstrual periods (effect indicator).

### Variables and data

All files were explored and data such as age and history of POR as well as laboratory results for levels of AMH, FSH, LH, and estradiol were gathered from each patient’s files. According to the POSEIDON criteria [3], each case fell under one of the following fourth groups (subgroups were not evaluated): Group 1: Age < 35 years, AMH ≥ 1.2 ng/mL; Group 2: Age ≥ 35 years, AMH ≥ 1.2 ng/mL; Group 3: Age < 35 years, AMH < 1.2 ng/mL; Group 4: Age ≥ 35 years, AMH < 1.2 ng/mL. The number of total oocytes was investigated by ultrasound evaluation and the type of oocytes (MI, MII, and GV) were assessed by an expert embryologist.

### Statistical analyses

All variables were extracted and re-checked by two authors (separately) and in case of any misinformation, the variable was checked by a third party involved in the preparation of original files. The continuous variables are presented as mean ± standard deviation (SD) or median ± interquartile range (IQR) for normally distributed and non-normally distributed variables respectively, as well and percentage (%) for categorical ones. In order to compare before and after the values, the paired T-test and the Wilcoxon test were used for parametric and non-parametric tests respectively. To assess differences between all the four POSEIDON groups, the Kruskal–Wallis test was used. For the association of categorical variables with an outcome, we used the chi-square/Fisher’s exact test. For statistical analyses, Statistical Package for the Social Sciences (SPSS) ver 25 (SPSS Inc, Chicago, IL, USA) was applied. Also, the statistical significance level was considered as *p* < 0.05.

## Results

In this study, a total number of 383 patients were evaluated of which 96 were enrolled and the other 287 were excluded according to the already mentioned criteria. The mean age of all involved cases was 38.30 ± 4.53 years. A total of 69/96 (71.9%) of patients were considered poor responders. Being a poor responder significantly differed among the groups (*P* = 0.002), however, the number of previous punctures did not (*P* = 0.755). Regarding the laboratory tests, their baseline levels of variables for all cases are provided in Table 1. According to the results, although treatment with PRP caused no improvement in the laboratory test results, it caused a significant increase in the total oocyte number as well as MII oocytes count (*P* < 0.001). The oocytes count of MI or GV and laboratory variables showed no statistically significant changes following the intervention Table 1. Moreover, 14 (14.6%) of total patients got pregnant by the end of the treatment and follow-up. Also, during the primary evaluations, it was shown that among the excluded cases due to the failure of follow-up for laboratory and oocyte evaluations, a total number of 28/287 (9.75%) cases got pregnant after PRP injection. The median age was of this group was 36 with an interquartile range (IQR) of 9 years. Their median ± IQR for primary AMH (ng/mL), LH (IU/L), FSH (IU/mL), and EST (pg/mL) levels were 1.09 ± 0.78, 4.07 ± 2.85, 6.17 ± 4.46, 50.3 ± 38.43, respectively. Also, the median number of oocytes was considered 3.5 ± 6.75. In this group, 5/28 (17.86%), 4/28 (14.28%), 7/28 (25.0%), and 12/28 (42.86%) of the cases were categorized into the POSEIDON groups of 1 to 4, respectively.

**Table 1:** Laboratory and oocyte-dependent characteristics before and after treatment with platelet-rich plasma (PRP) in the whole population

Variables	Before PRP (Median ± IQR)	After PRP (Median ± IQR)	*P*-value
LH	2.80 ± 1.93	2.88 ± 1.76	0.962
FSH	6.21 ± 4.48	6.41 ± 3.72	0.059
Estradiol	51.00 ± 34.10	50.00 ± 32.68	0.771
AMH	0.72 ± 0.87	0.73 ± 1.04	0.905
Total ovocyte number	2.00 ± 3.00	3.00 ± 6.00	** < 0.001**
GV ovocyte number	0.00 ± 0.00	0.00 ± 0.00	0.604
MI ovocyte number	0.00 ± 0.00	0.00 ± 0.00	0.210
MII ovocyte number	1.00 ± 3.00	3.00 ± 5.00	** < 0.001**

*LH* Luteinizing hormone, *FSH* Follicle-stimulating hormone, *AMH* Anti-Mullerian hormone

*P*-values have been calculated by Wilcoxon rank test

The main aim of this study was to investigate the oocytes dependent and laboratory variables in the POSEIDON groups following the PRP treatment. According to the age and AMH levels, the patients were divided into the four already mentioned POSEIDON groups with the following prevalence: Group 1: 7/96 (7.3%), Group 2: 17/96 (17.7%), Group 3: 16/96 (16.7%), and Group 4: 56/96 (58.3%). Obviously, these groups had statistically significant differences in age and AMH levels on baseline levels (*P* < 0.001), however, the other laboratory variables didn’t show such results. In the post-treatment laboratory test results, the baseline levels of FSH (*P* = 0.028) and AMH (*P* < 0.001) differed significantly among the four groups. Other than AMH in the POSEIDON group 2, no other laboratory variable expressed any significant changes following the PRP treatment compared to their baseline levels (Table 2).

**Table 2:** Laboratory characteristics before and after treatment with platelet-rich plasma (PRP) in for POSEIDON groups

Variables		POSEIDON 1 Median ± IQR	POSEIDON 2 Median ± IQR	POSEIDON 3 Median ± IQR	POSEIDON 4 Median ± IQR	*P*-value
Age (years)		32.00 ± 3.00	37.00 ± 8.00	33.00 ± 2.00	40.00 ± 5.00	** < 0.001**
Poor responder (%)		2/7 (28.6%)	8/17 (47.1%)	12/16 (75%)	47/56 (83.9%)	**0.002**
Number of previous punctures		3 ± 2	3 ± 1	2.5 ± 1	2.00 ± 2.00	0.755
LH (IU/L)	Before PRP	1.87 ± 1.50	2.35 ± 2.82	2.51 ± 1.30	3.02 ± 1.75	0.160
	After PRP	2.08 ± 1.43	2.98 ± 1.68	2.82 ± 2.20	3.05 ± 2.29	0.198
FSH (IU/mL)	Before PRP	4.27 ± 1.73	5.79 ± 2.65	6.08 ± 5.19	6.66 ± 5.11	0.054
	After PRP	5.15 ± 1.63	5.60 ± 3.20	6.85 ± 4.23	7.21 ± 4.04	**0.028**
Estradiol (pg/mL)	Before PRP	31.00 ± 55.9	46.00 ± 16.65	57.15 ± 40.10	51.60 ± 34.10	0.576
	After PRP	55.80 ± 20.30	51.70 ± 23.30	62.00 ± 45.68	45.00 ± 33.38	0.342
AMH (ng/mL)	Before PRP	1.75 ± 2.07	1.68 ± 0.98	0.6 ± 0.45	0.46 ± 0.51	** < 0.001**
	After PRP	1.85 ± 2.56	1.53 ± 1.03 *	0.64 ± 0.68	0.50 ± 0.56	** < 0.001**

*IQR* Interquartile range, *LH* Luteinizing hormone, *FSH* Follicle-stimulating hormone, *AMH* Anti-Mullerian hormone

^*^: *p*-value < 0.05, **: *p*-value < 0.01, and ***: *p*-value < 0.001 all compare to the before level

For all the POSEIDON groups and among the studied oocyte-dependent variables, the count of total and GV oocytes was significantly different (*P* = 0.004 and 0.003 respectively) among the groups before the intervention. Following the treatment, these variables including total (*P* < 0.001), MI (*P* = 0.004), and MII (*P* = 0.001) oocytes count significantly differed among the groups (Table 3). Also, after treatment with PRP, all the POSEIDON groups exhibited a statistically significant increase in their total number of oocytes compared to the baseline value (PSEIDONG groups 1, 2, 3, and 4 had *P*-values of 0.018, 0.026, 0.047, and < 0.001 respectively). For the MII oocytes, it was shown that the POSEIDON groups 1, 3, and 4 had statistically significant increase in the count with the *P*-value of 0.018, 0.026, and < 0.001, respectively. Other details of the results are provided in Table 3. Finally, the number of fetuses and clinical pregnancy significantly differed among the groups (*P* = 0.005 and *P* = 0.002 respectively) with group 1 POSEIDON having the highest numbers compared to the others (5/7 or 71.42% for pregnancy and 17 ± 13 (IQR) for the number of the fetuses).

**Table 3:** Oocytes characteristic features before and after treatment with platelet-rich plasma (PRP) in for POSEIDON groups

Variables		POSEIDON 1 Median ± IQR	POSEIDON 2 Median ± IQR	POSEIDON 3 Median ± IQR	POSEIDON 4 Median ± IQR	*P*-value
Total Ovocyte number	Before PRP	4.00 ± 2.00	2.00 ± 3.00	1.00 ± 3.00	1.00 ± 1.00	**0.003**^╪^
	After PRP	9.00 ± 5.00 *	5.00 ± 5.00 *	2.00 ± 7.00 *	3.00 ± 4.00 ***	** < 0.001**^╪^
GV Ovocyte number	Before PRP	1.00 ± 2.00	0.00 ± 0.5	0.00 ± 1.00	0.00 ± 0.00	**0.003**^╪^
	After PRP	0.00 ± 0.00	0.00 ± 1.00	0.00 ± 0.75	0.00 ± 0.00	0.085^┼^
MI Ovocyte number	Before PRP	0.00 ± 0.00	0.00 ± 0.00	0.00 ± 0.00	0.00 ± 0.00	0.175^┼^
	After PRP	1.00 ± 1.00	0.00 ± 1.00	0.00 ± 0.00	0.00 ± 0.00	**0.007**^┼^
MII Ovocyte number	Before PRP	2.00 ± 3.00	2.00 ± 3.00	1.00 ± 3.00	1.00 ± 2.00	0.052^╪^
	After PRP	8.00 ± 3.00 *	4.00 ± 3.50	2. .00 ± 6.50 *	2.00 ± 3.75 ***	**0.001**^╪^
Fetus number		17.00 ± 13.00	4.00 ± 5.00	3.00 ± 8.75	2.00 ± 6.00	**0.005**^╪^
Pregnancy		5/7 (71.42%)	2/17 (11.76%)	1/16 (6.25%)	6/56 (10.71%)	**0.002**^┼^

^*^: *p*-value < 0.05, **: *p*-value < 0.01, and ***: *p*-value < 0.001 all compare to the before level. ^┼^: Fisher exact test. ^╪^: Kruskal Wallis

## Discussion

The current study investigated the possible potentials of intra-ovarian PRP administration on the POR patients. According to the results and following single session of the intervention, total oocytes count showed a statistically significant increase in all POSEIDON groups with more notable changes in group 4. For the MI, MII, and GV oocytes, only MII ones experienced a significant increase in their count following PRP administration (but not in the group 2; Age ≥ 35 years, AMH ≥ 1.2). Interestingly, this study showed that 9.75% of women with no ART got pregnant following a single session of PRP injection. However, it was less than 14.75% for those cases undergone ART. On the other hand, except for AMH in group 2 POSEIDON (17 patients), none of the other laboratory variables experienced a significant change in any of the POSEIDON groups. This result showed that the changes observed in the outcomes of patients are more likely achieved through a non-hormonal pathway(s).

PRP is an autologous product and has a notable amount of α-granules releasing their many growth factors following their degradation. These factors are basic fibroblast growth factor (bFGF), fibroblast growth factor (FGF), epidermal growth factor (EGF), vascular endothelial growth factor (VEGF), insulin-like growth factor-1 (IGF-1), platelet-derived growth factors (PDGF-AA, PDGF-AB, and PDGF-BB), and transforming growth factors β1 and 2 (TGF-β1 and 2) which are the key role players of angiogenesis and regeneration [20–22]. As Hajipour et al. have mentioned in their systematic review, growth factors in the PRP could affect different characteristic features of oocytes to eventually increase survival rate of follicles compared to the controls [20].

Other than the mentioned applications of PRP, it has been a rather long-used product in the field of infertility as well. PRP is also been suggested administration in some endometrial and ovarian complications [20]. Cakiroglu et al., investigated the results of intra-ovarian PRP administration on primary ovarian insufficiency. They found that PRP treatment could lead to increased antral follicle count as well as AMH but not FSH. As they have stated, 201 of 311 (64.8%) PRP treated women developed antral follicle(s). Finally, they have concluded their observation as improved ovarian function after the PRP treatment [23].

Also, recently, a retrospective study evaluated the effects of growth hormone (GH) treatment on cases with POR candidates for intracytoplasmic sperm injection (ICS)/IVF according to the POSEIDON groups (only those aged > 35 years). They have shown that number of oocytes retrieved, transferrable embryos, and good quality embryos were not significantly different in GH treated and control groups. According to their results, only individuals treated with GH in group 4 of POSEIDON had significantly higher good-quality embryos compared to the non-treated group [24].

This study has included notable numbers of women with the diagnosis of POR which is much higher than similar studies [15]. Also, one of the strength points of this study was the assessment of all laboratory results with a single kit, device, and operator which greatly limits possible errors.

## Conclusion

An intra-ovarian injection of PRP in women diagnosed with POR showed results in favor of improved ovarian function including total oocyte number and especially MII oocytes. On the other hand, AMH (other than POSEIDON group 2), FSH, LH, and estradiol did not significantly change. This might be a clue to how PRP therapy in these patients did not exploited the hormonal pathways and there might be other mechanisms involved such as angiogenesis. All and all, it seems that PRP could be a proper treatment candidate for the patients with POR who show resistance to the other treatments such as hormonal therapies. The authors of this study strongly suggest further investigations on this hot topic to clarify the potential of intra-ovarian PRP therapy as well as its exact mechanism(s).

## Data Availability

Data would be available at online requests.

## References

[1] Coccia ME, Rizzello F (2008). Ovarian reserve. Ann N Y Acad Sci.

[2] Surrey ES, Schoolcraft WB (2000). Evaluating strategies for improving ovarian response of the poor responder undergoing assisted reproductive techniques. Fertil Steril.

[3] Alviggi C, Andersen CY, Buehler K, Conforti A, De Placido G, Esteves SC (2016). A new more detailed stratification of low responders to ovarian stimulation: From a poor ovarian response to a low prognosis concept. Fertil Steril.

[4] Grisendi V, Mastellari E, La Marca A (2019). Ovarian reserve markers to identify poor responders in the context of Poseidon classification. Front Endocrinol.

[5] Ferraretti AP, La Marca A, Fauser BC, Tarlatzis B, Nargund G, Gianaroli L (2011). ESHRE consensus on the definition of ‘poor response’ to ovarian stimulation for in vitro fertilization: the Bologna criteria. Hum Reprod.

[6] Esteves SC, Alviggi C, Humaidan P, Fischer R, Andersen CY, Conforti A (2019). The POSEIDON criteria and its measure of success through the eyes of clinicians and embryologists. Front Endocrinol.

[7] Mohammadi S, Nasiri S, Mohammadi MH, Malek Mohammadi A, Nikbakht M, Zahed Panah M (2017). Evaluation of platelet-rich plasma gel potential in acceleration of wound healing duration in patients underwent pilonidal sinus surgery: A randomized controlled parallel clinical trial. Transfus Apher Sci.

[8] Mohammadi MH, Molavi B, Mohammadi S, Nikbakht M, Mohammadi AM, Mostafaei S (2017). Evaluation of wound healing in diabetic foot ulcer using platelet-rich plasma gel: A single-arm clinical trial. Transfus Apher Sci.

[9] Serra-Mestre JM, Serra-Renom JM, Martinez L, Almadori A, D'Andrea F (2014). Platelet-rich plasma mixed-fat grafting: A reasonable prosurvival strategy for fat grafts?. Aesthetic Plast Surg.

[10] de Vos RJ, Weir A, van Schie HT, Bierma-Zeinstra SM, Verhaar JA, Weinans H (2010). Platelet-rich plasma injection for chronic Achilles tendinopathy: A randomized controlled trial. JAMA.

[11] Farimani M, Bahmanzadeh M, Poorolajal J (2016). A new approach using autologous platelet-rich plasma (PRP) to treat infertility and to improve population replacement rate. J RE Health Sci.

[12] Farimani M, Heshmati S, Poorolajal J, Bahmanzadeh M (2019). A report on three live births in women with poor ovarian response following intra-ovarian injection of platelet-rich plasma (PRP). Mol Biol Rep.

[13] Bos-Mikich A, de Oliveira R, Frantz N (2018). Platelet-rich plasma therapy and reproductive medicine. J Assist Reprod Genet.

[14] Cakiroglu Y, Saltik A, Yuceturk A, Karaosmanoglu O, Kopuk SY, Scott RT (2020). Effects of intraovarian injection of autologous platelet rich plasma on ovarian reserve and IVF outcome parameters in women with primary ovarian insufficiency. Aging.

[15] Aflatoonian A, Lotfi M, Saeed L, Tabibnejad N (2021). Effects of intraovarian injection of autologous platelet-rich plasma on ovarian rejuvenation in poor responders and women with primary ovarian insufficiency. Reprod Sci.

[16] Farimani M, Poorolajal J, Rabiee S, Bahmanzadeh M (2017). Successful pregnancy and live birth after intrauterine administration of autologous platelet-rich plasma in a woman with recurrent implantation failure: A case report. Int J Reproduct Biomed.

[17] Younis JS, Ben-Ami M, Ben-Shlomo I (2015). The Bologna criteria for poor ovarian response: A contemporary critical appraisal. Journal of ovarian research.

[18] Mohamadi S, Norooznezhad AH, Mostafaei S, Nikbakht M, Nassiri S, Safar H (2019). A randomized controlled trial of effectiveness of platelet-rich plasma gel and regular dressing on wound healing time in pilonidal sinus surgery: Role of different affecting factors. Biomed J.

[19] Kuang Y, Chen Q, Hong Q, Lyu Q, Ai A, Fu Y (2014). Double stimulations during the follicular and luteal phases of poor responders in IVF/ICSI programmes (Shanghai protocol). Reprod Biomed Online.

[20] Hajipour H, Farzadi L, Latifi Z, Keyhanvar N, Navali N, Fattahi A (2021). An update on platelet-rich plasma (PRP) therapy in endometrium and ovary related infertilities: clinical and molecular aspects. Syst Biol Reprod Med..

[21] Mostafaei S, Norooznezhad F, Mohammadi S, Norooznezhad AH (2017). Effectiveness of platelet-rich plasma therapy in wound healing of pilonidal sinus surgery: A comprehensive systematic review and meta-analysis. Wound Repair Regen.

[22] Lacci KM, Dardik A (2010). Platelet-rich plasma: Support for its use in wound healing. Yale J Biol Med.

[23] Sills ES, Rickers NS, Li X, Palermo GD (2018). First data on in vitro fertilization and blastocyst formation after intraovarian injection of calcium gluconate-activated autologous platelet rich plasma. Gynecol Endocrinol.

[24] Cai MH, Gao LZ, Liang XY, Fang C, Wu YQ, Yang X (2019). The effect of growth hormone on the clinical outcomes of poor ovarian reserve patients undergoing in vitro fertilization/intracytoplasmic sperm injection treatment: A retrospective study based on POSEIDON criteria. Front Endocrinol (Lausanne).

