# Correction to: Evaluation of intra-ovarian platelet-rich plasma administration on oocytes-dependent variables in patients with poor ovarian response: a retrospective study according to the POSEIDON criteria

**DOI:** 10.1186/s12958-021-00849-3

**Published:** 2021-11-18

**Authors:** Marzieh Farimani, Arash Nazari, Shahrzad Mohammadi, Roghayeh Anvari Aliabad

**Affiliations:** 1 Endometrium and Endometriosis Research Center, Hamadan University of Medical Sciences, Hamadan, Iran; 2 School of Medicine, Hamadan University of Medical Sciences, Hamadan, Iran; 3 School of Medicine, Iran University of Medical Sciences, Tehran, Iran; 4 Department of Gynecology, School of Medicine, Hamadan University of Medical Sciences, Hamadan, Iran; 5 Endometrium and Endometriosis Research Center, Fatemieh Hospital, Pasdaran Street, P.O. Box, Hamadan, 89971-65177 Iran

**Correction to: Reprod Biol Endocrinol 19, 137 (2021)**

**https://doi.org/10.1186/s12958-021-00826-w**

Following publication of the original article [1], the authors reported two errors. In the introduction, "A report on 23 women with primary ovarian insufficiency has shown that PRP could be a proper treatment option for these patients." should be changed to "A report on 23 women (of 311) with primary ovarian insufficiency who conceived spontaneously has shown that PRP could be a proper treatment option for these patients."

The other one is that the reference number 23 (Sills ES, Rickers NS, Li X, Palermo GD. First data on in vitro fertilization and blastocyst formation after intraovarian injection of calcium gluconate-activated autologous platelet rich plasma. Gynecol Endocrinol. 2018;34:756–760. doi: 10.1080/09513590.2018.1445219) should be updated as following:

Cakiroglu Y, Saltik A, Yuceturk A, Karaosmanoglu O, Kopuk SY, Scott RT, et al. Effects of intraovarian injection of autologous platelet rich plasma on ovarian reserve and IVF outcome parameters in women with primary ovarian insufficiency. Aging. 2020;12:10211–22. doi: 10.18632/aging.103403.

The original article [1] has been updated.
